# In the Shadows of the Congo Basin Forest, Elephants Fall to the Illegal Ivory Trade

**DOI:** 10.1371/journal.pbio.0050115

**Published:** 2007-04-03

**Authors:** Liza Gross

The 1980s were not kind to African elephants. Poachers committed brutal acts in the pursuit of tusks to feed the human hunger for ivory. Gruesome images of the carnage they left behind—mutilated corpses sprawled in twisted repose, attended by bereft companions and bewildered orphans—helped document the precipitous collapse of Africa’s elephant populations. Between 1970 and 1989, as global demand for ivory grew and poachers traded traditional hunting methods for AK-47s and elephant guns, an estimated three-quarters of a million elephants were killed, leaving just 600,000 survivors. Much of Africa, once home to as many as 10 million elephants, had turned into an elephant graveyard.

This catastrophic decline, coupled with public outcry at the elephants’ cruel fate, led to a ban on the international ivory trade, after the Convention on International Trade in Endangered Species of Wild Fauna and Flora (CITES) placed the African elephant on its most critically endangered list, Appendix I. The ban, which led to more aggressive anti-poaching campaigns and increased investment in wildlife protection, set African elephants on the road to recovery. But a new study from Stephen Blake, Samantha Strindberg, Fiona Maisels, and colleagues warns that while savannah elephants may indeed be rebounding—in part because they live in countries with long histories of wildlife management, where protection is facilitated by open plains habitats and usually good infrastructure—their forest relatives, hidden in the Congo Basin rainforests, still face intense poaching pressure.

Humans have exploited elephants’ ivory tusks for profit ever since ancient civilizations recognized the beauty and commercial potential of ivory. Despite increased awareness about the bloody legacy of the illegal ivory trade, consumers outside Africa, particularly in Asia, continue to buy ivory. And despite the 1989 ban, the status and management of African elephants remain precarious, except in southern Africa, where effective management of the savannah elephant has ensured its protection. Countries in southern Africa oppose the Appendix I listing and trade ban, arguing that their savannah elephants have either rebounded or were never threatened, thanks to good management. They want to sell their ivory stockpiles as reward for their success and use the proceeds to underwrite their conservation programs. (Savannah populations appear mostly stable and relatively protected from poachers—though a 2006 report found evidence of increased poaching in Mozambique and Angola, the only sub-Saharan nation that is not a member of CITES.) CITES approved one-time stockpile sales in 1997 and in 2002; though the 2002 sale has not yet proceeded, pending the completion of necessary monitoring commitments, it may be approved by CITES in June.

**Figure:**
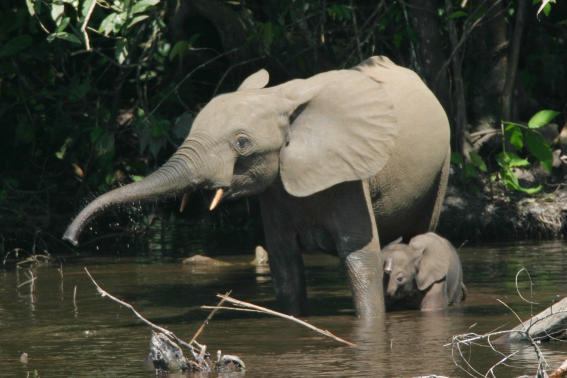
Forest elephants in the Mbeli River, Nouabalé-Ndoki National Park, Congo. Despite a near universal ban on trade in ivory, poaching is placing the African forest elephant—distinct from the savannah elephant—in serious jeopardy. (Image: Thomas Breuer)

While the life history and conservation status of savannah elephants are well known, relatively little is known about the biology of forest elephants, which scientists suspect may be a distinct species uniquely adapted to Africa’s dense rainforests. And because central African nations—where ivory poaching persists—have far less capacity for elephant management than southern African nations, conservationists fear that legalizing even limited trade in ivory from southern African elephants will place the forest elephant in serious jeopardy.

Unlike most eastern and southern African nations, which have thriving wildlife-based tourism industries and the funding and infrastructure to facilitate elephant protection, Congo Basin forest nations have crumbling infrastructure, poorly supported wildlife departments, and vast areas accessible only by foot. In some nations, high levels of corruption thwart wildlife management, while in the Democratic Republic of Congo, Congo, and the Central African Republic, high-intensity civil conflicts have interfered with management and led to accelerated poaching, often organized by the military. In nations without the law enforcement capacity to catch and prosecute ivory poachers, poaching levels appear to fluctuate with market demand and price. Kenya, which saw rampant elephant poaching in the 1980s, and countries in West and Central Africa, the heart of forest elephant habitat, continue to support the Appendix 1 listing of all African elephants. They worry that even one-off sales will stimulate global demand for ivory, expand the black market for ivory, and increase the incentive to poach.

Devising a rational policy for managing elephants that balances the rights of local governments against wildlife conservation requires accurate measures of elephant abundance and distribution and rates of illegal killing across the continent. In savannahs, elephant counts usually involve flying over the plains to count both live individuals and carcasses. But tracking elephants and poachers through the vast, often swampy equatorial rainforest must be done on foot and presents significant methodological and logistical challenges. The last regional forest elephant survey, in 1989, estimated that about 172,000 forest elephants lived in the Congo Basin—suggesting that forest elephants accounted for nearly a third of Africa’s elephants at that time. Forest elephant distribution, the survey found, was primarily affected by the presence of humans and roads.

With logging and road-building in the Congo Basin projected to increase dramatically, Blake et al. set out to chart the abundance and distribution of forest elephants across the Congo Basin. Working in conjunction with the CITES Monitoring of the Illegal Killing of Elephants (MIKE) program, the researchers systematically surveyed six protected MIKE sites, national parks expected to have significant forest elephant populations. Between 2003 and 2005, they walked through “some of the most remote and difficult terrain in forested Africa” to survey over 60,000 square kilometers for signs of elephants (dung piles) and humans (machete cuts, shotgun cartridges, snares, and hunting camps), to collect data on the abundance, distribution, and illegal killing of forest elephants (indicated by carcasses found with gunshot holes in the skull and removed tusks, or by elephant meat on smoking racks in active camps). Their analysis included data collected during a “megatransect,” a continuous foot survey through 2,000 kilometers of “the most remote forest blocks in Africa,” between northeastern Congo and southwestern Gabon.

As expected, illegal killing and other human incursions played a profound role in shaping forest elephant distribution and abundance. Altogether, the researchers found 53 confirmed elephant poaching camps; they found poached elephant carcasses, with tusks removed, in every protected MIKE site. The probability of encountering elephants increased as one traveled away from the nearest major road, while the probability of detecting human signs decreased. The likelihood of discovering poached elephant carcasses also decreased with distance from the nearest road; no poached carcasses were found farther than 45 kilometers from the nearest road.

In Salonga National Park—the largest forested national park in Africa, and a UNESCO World Heritage Site in the remote heart of the Democratic Republic of Congo—Blake et al. report as few as 1,900 elephants remain. Only two national parks had a mean estimated density of more than one elephant per square kilometer, Minkébé and Odzala-Koukoua, and these sites contain the largest tracts of remote wilderness left in the entire Congo Basin. The overwhelming trend from both the MIKE surveys and the megatransect was that elephants were more abundant with increasing distance from a road and decreasing evidence of humans.

How could Salonga National Park, with three times the area of any other park surveyed, have so few elephants? Most likely because the park is divided into two sections, and is crisscrossed with old roads and navigable rivers, placing nearly half of its area within ten kilometers of an access point. In contrast, the two parks with remote areas over 60 kilometers from the nearest roads had elephant densities over ten times that of Salonga.

Since the megatransect survey included both protected and unprotected areas over a large geographical scale, the researchers could determine what effects, if any, protected status has on the number and distribution of forest elephants and on the presence of humans. As in the analysis of the protected MIKE sites, the probability of encountering elephants increased with distance from a road, but at any distance from a road the probability of encountering elephants was higher in protected areas than in forest outside of protected areas. Protected areas, even those with limited management capacity, have a positive influence by attenuating rates of illegal killing.

Overall, these results show that forest elephants are in deep trouble—their range and numbers are shrinking as poachers continue to kill them for their tusks (and probably their meat). As logging road infrastructure explodes across the Congo Basin, the area of remote wilderness is decreasing rapidly, allowing poachers access to the last herds in the depths of the forest. Large areas of wilderness are critical for the survival of forest elephants, and important if human–elephant conflict is to be minimized, yet they are disappearing fast.

Given that this study describes the situation in and around well-established protected areas and remote forest, the researchers point out that the conservation status of forest elephants is probably “considerably worse” in the rest of the Congo Basin, where wildlife management resources are scarce. Legalizing the ivory trade based on the recovery of savannah elephants will only accelerate the forest elephants’ decline, unless a change in trade status of southern African elephants comes with a strong commitment from CITES and ivory-trading nations to work with central African nations to reinforce elephant management capacity.

What can be done to save the forest elephant? Given that protected areas tend to have more elephants and less people, the preservation of the forest elephant depends on improving management capacity in large, remote, well-managed national parks throughout their range in the Congo Basin. Preventing further declines will require beefed-up law enforcement to stop poaching and rein in the illegal ivory trade, carefully regulated road-building to preserve contiguous forest, and collaborations with logging and mining companies to reduce their impacts on protected areas and to improve ecosystem management and elephant protection in their concessions. Ultimately, consumers of unsustainable and illegally traded ivory must be educated to understand that their actions leave bloody, rotting carcasses in the depths of the forest. Illegal trade provides precious little benefit to the usually poor poacher, who may kill a six-ton elephant with 30-kilogram tusks worth thousands of dollars on the international market for the “salary” of a pair of shorts and a packet of cigarettes from an ivory-trading middleman.

While the elephant clearly awakens the darker side of humanity, willing to lay waste a giant beast for the sake of its tusk, one can only hope that it can also inspire the human capacity for wisdom—and the understanding that the world would be a poorer place without every incarnation of what Donne called “nature’s great master-peece.” For more information on African elephants, visit http://www.wcs.org/international/Africa/africanelephants or http://www.iucn.org/themes/ssc/sgs/afesg/aed/aesr2002.html.

